# Pediatric Bilateral Hypoplastic Kidney Complicated With C1q Nephropathy: A Case Report

**DOI:** 10.7759/cureus.63923

**Published:** 2024-07-05

**Authors:** Hiroaki Kanai, Emi Sawanobori, Anna Kobayashi, Miwa Goto

**Affiliations:** 1 Department of Pediatrics, Suwa Central Hospital, Chino, JPN; 2 Department of Pediatrics, Faculty of Medicine, University of Yamanashi, Chuo, JPN

**Keywords:** enalapril, kidney biopsy, mesangial proliferative glomerulonephritis, c1q nephropathy, hypoplastic kidney

## Abstract

Progressive kidney dysfunction is often observed in children with bilateral hypoplastic kidneys. While glomerulopathy can exacerbate hypoplastic kidney progression, only IgA nephropathy and post-streptococcal acute glomerulonephritis have been noted in such cases. Herein, we present a case of a four-year-old female patient with bilateral hypoplastic kidney, kidney dysfunction, and significant proteinuria (urinary protein/creatinine ratio > 1 g/gCr), prompting referral owing to persistent hematuria since two years of age. Enalapril was initiated; however, urinary findings exhibited no improvement despite stable symptoms and kidney function. Subsequently, a kidney biopsy was performed at six years of age, and C1q nephropathy was diagnosed. Given the presence of only mild mesangial proliferation, steroids were not administered; enalapril treatment was continued. By seven years of age, the patient's hematuria had resolved, and proteinuria levels had decreased. On the latest follow-up at 12 years of age, kidney function was preserved with only mild proteinuria. This case report highlights the favorable prognosis of asymptomatic C1q nephropathy characterized by mild mesangial proliferation, even in patients with hypoplastic kidneys, renal dysfunction, and significant proteinuria. It emphasizes the significance of timely pathological evaluation for guiding appropriate interventions in such patients.

## Introduction

Children with hypoplastic kidneys, particularly those with bilateral involvement, often experience a progressive decline in kidney function [1,2]. Notably, a few cases of IgA nephropathy (IgAN) and post-streptococcal acute glomerulonephritis (PSAGN) have been reported in individuals with low nephron numbers (such as hypoplastic kidney and solitary kidney). These studies suggest that concomitant nephropathy can synergistically worsen kidney dysfunction and result in rapid progression [3-7]. However, to the best of our knowledge, no studies of other glomerulopathies are available in this context. To the best of our knowledge, this report describes the first pediatric case of C1q nephropathy (C1qN; a rare form of chronic glomerulonephritis (GN)) in a child with bilateral hypoplastic kidney.

## Case presentation

A four-year-old female patient with bilateral hypoplastic kidney was referred to our hospital for evaluation of massive proteinuria with microscopic hematuria. She was born at 38 weeks and three days of gestation via normal delivery, with a birth weight of 2,460 g. At two days of age, the patient was admitted for dehydration and was subsequently diagnosed with bilateral hypoplastic kidney on ultrasonography. She experienced no episodes of urinary tract infection. She underwent regular follow-ups, maintaining an estimated glomerular filtration rate (eGFR) of 60 mL/min/1.73 m^2^ and a urinary protein/creatinine ratio (UPCR) exceeding 1 g/gCr. However, at two years of age, she developed persistent microscopic hematuria.

**Figure 1:**
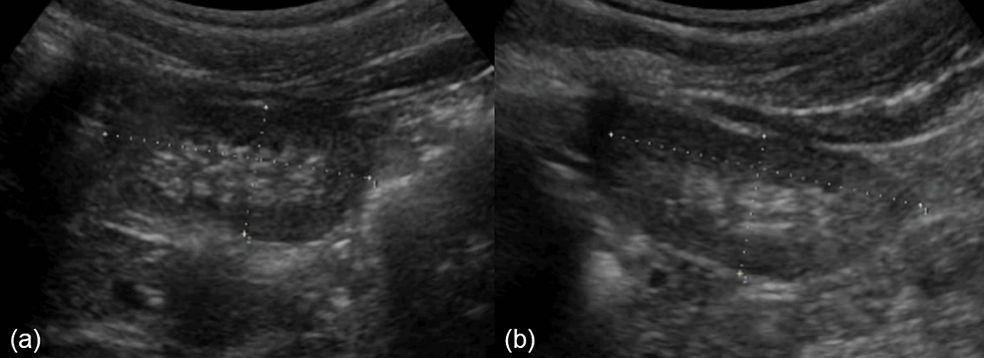
Ultrasonography findings. Increased echogenicity of the renal cortex and bilateral hypoplastic kidney with (a) a right renal length of 5.0 cm and (b) a left renal length of 5.3 cm (-2 standard deviations for her height of 5.7 cm).

Upon referral to our hospital, her blood pressure and physical examination findings were unremarkable. Laboratory findings indicated no hypoalbuminemia (serum albumin level, 3.8 g/dL), mild kidney dysfunction (serum creatinine (Cr) level, 0.52 mg/dL; eGFR, 62.5 mL/min/1.73 m^2^), massive proteinuria (UPCR, 1.4 g/gCr), and microscopic hematuria (10-19 red blood cells per high power field), with dysmorphic red blood cells [8]. The test results for hepatitis B surface antigen, anti-hepatitis C antibody, and antinuclear antibody were negative, with no evidence of hypocomplementemia. Additionally, ultrasonography findings indicated increased echogenicity of the renal cortex, with a right renal length of 5.0 cm and a left renal length of 5.3 cm (-2 standard deviations (SDs) for her height of 5.7 cm [9]) (Figure 1). Given these findings, a pathological evaluation was deemed necessary. However, initial consent for the biopsy could not be obtained; therefore, her condition was managed with enalapril treatment. Despite no worsening kidney function or development of nephrotic syndrome, her urine test results did not improve. A percutaneous kidney biopsy was eventually performed at the age of six years.

**Figure 2:**
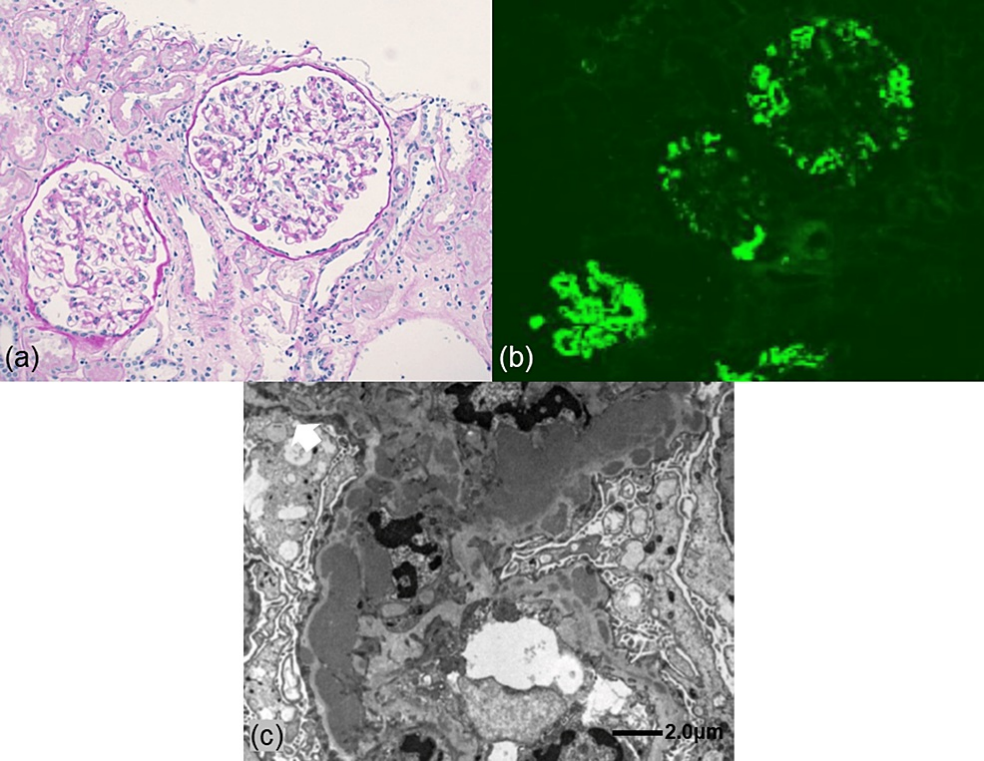
Kidney biopsy findings. (a) Periodic acid-Schiff staining: focal segmental mesangial proliferation. Crescents, sclerosis, interstitial lesions, and glomerular hypertrophy are absent (original magnification, ×200). (b) Immunofluorescence showing predominant granular deposits of C1q (2+) in the mesangium and along the glomerular capillary walls. (c) Electron microscopy showing electron-dense deposits in the mesangium.

Light microscopy examination revealed 14 glomeruli, with focal segmental mesangial proliferation. However, infiltration of polymorphonuclear leukocytes in glomerular capillaries, crescents, glomerulosclerosis, glomerular enlargement, and interstitial lesions, including tubular hypertrophy, were absent. In addition, no findings were suggestive of dysplasia of multiple cysts, primitive duct surrounded by undifferentiated stroma, ectopic cartilage, and cystic tubule dilatations. Immunofluorescence revealed predominant mesangial staining for C1q (2+) and lesser staining for IgG (1+), IgM (1+), C3 (1+), C4 (1+), and IgA (+/-). Electron microscopy revealed electron-dense deposits in the mesangium. These findings collectively culminated in the diagnosis of C1qN (Figure 2).

**Figure 3:**
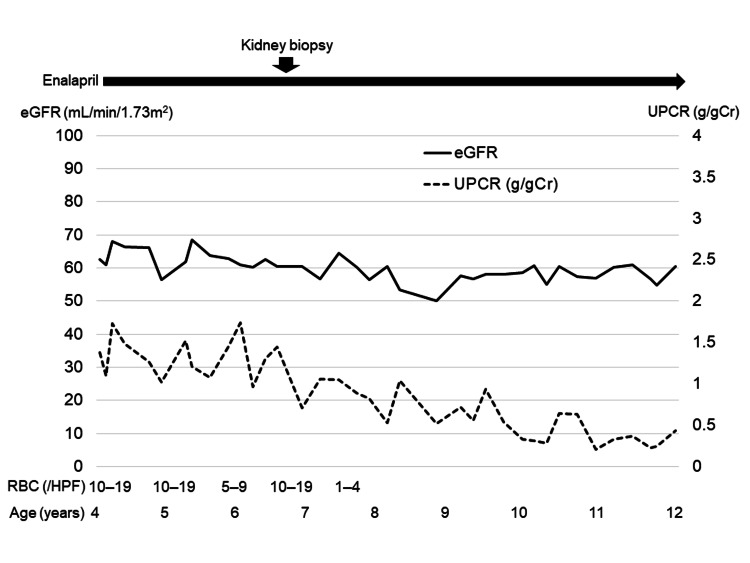
Clinical course of the patient. The patient was referred at four years of age for evaluation of massive proteinuria with hematuria. Although enalapril was initiated, urinary findings did not improve. A kidney biopsy was performed at six years of age, leading to the diagnosis of C1q nephropathy. Hematuria was eventually resolved, and proteinuria levels decreased by seven years of age. On her latest follow-up at 12 years of age, kidney function is preserved with mild proteinuria. eGFR: estimated glomerular filtration rate; UPCR: urine protein/creatinine ratio; RBC: red blood cells; HPF: high power field.

Considering the patient’s asymptomatic state and pathological findings of mild mesangial proliferation, steroids were withheld, and only enalapril administration was continued. On subsequent follow-up visits, the patient’s hematuria had resolved, and proteinuria had decreased by the age of seven years. At 11 years of age, an ultrasound scan indicated a right renal length of 7.3 cm and a left renal length of 7.5 cm (-2 SDs for her height of 7.7 cm [9]). The patient was maintained on enalapril (6 mg/day, 0.15 mg/kg), and her latest follow-up at the age of 12 years revealed preserved kidney function (serum Cr, 0.88 mg/dL; eGFR, 58.8 mL/min/m^2^) and mild proteinuria (UPCR, 0.40 g/gCr), without hypoalbuminemia (serum albumin, 3.8 g/dL) and hematuria (Figure 3).

## Discussion

C1qN was diagnosed on the basis of histological findings exhibiting dominant or co-dominant staining for C1q, confirmation of mesangial deposits by electron microscopy, and absence of clinical or serologic evidence of systemic lupus erythematosus. This is a rare form of glomerulopathy that presents with diverse histological variants, including minimal change disease, focal segmental glomerulosclerosis (FSGS), and immune complex nephritis such as mesangial proliferative GN [10,11]. Similarly, its clinical presentation is diverse, ranging from asymptomatic hematuria and proteinuria to nephrotic syndrome [10,11]. Consequently, treatment strategies are usually contingent on the patient’s clinical symptoms and pathological findings [11,12]. Although the mainstay management of nephrotic cases includes steroids with or without immunosuppressants, the treatment approaches for asymptomatic cases vary [12,13]. In most asymptomatic cases in adults and children, minor glomerular abnormalities or proliferative GN are not treated with steroids due to the possible resolution of urinary abnormalities and preservation of renal function [11,12]. Conversely, in such cases, FSGS is associated with poorer outcomes, even when steroids are administered [12,13].

The renal prognosis for hypoplastic kidneys is typically unfavorable (particularly in bilateral cases), with many children experiencing progressive kidney decline that can ultimately lead to end-stage renal failure [2]. This decline is primarily attributed to a reduction in nephron numbers, resulting in increased glomerular filtration, intraglomerular hypertension, and compensatory hypertrophy, ultimately leading to secondary FSGS [14]. Therefore, kidney biopsy is not routinely indicated in hypoplastic kidneys even in the presence of proteinuria since pathological evaluation does not typically influence the treatment strategy. However, the presence of both hematuria and proteinuria significantly increases the likelihood of GN, warranting a pathological evaluation.

Our patient presented with bilateral hypoplastic kidney at the age of four years, persistent hematuria since the age of two years, and massive proteinuria since birth. Given these clinical findings, we determined that a pathological evaluation was necessary. A kidney biopsy was eventually performed at six years of age and led to the diagnosis of C1qN. Although the exact onset of C1qN was uncertain, we estimated the onset to be at two years of age considering the pathological findings of mesangial proliferative GN similar to IgAN and hematuria onset. Notably, findings from the kidney biopsy performed over four years after the presumed onset revealed only mild mesangial proliferation, with no signs of sclerosis, interstitial lesions, or crescents. This suggested a mild C1qN course, possibly attributable to the reduced intraglomerular pressure effect of enalapril despite the patient’s condition.

Steroid use was not initiated owing to stable renal function and mild pathological findings in our patient. However, renal prognosis, in this case, was expected to be poor based on a lack of accurate information on the long-term renal prognosis of pediatric C1qN, and several studies suggest that renal function in patients with low nephron numbers can worsen in the context of concurrent nephropathies [3-7]. Fortunately, the patient’s hematuria resolved, and proteinuria levels were decreased following the continuation of enalapril treatment within 10 months of the biopsy. Moreover, renal function remained stable at approximately 10 years since onset, suggesting a potential renoprotective effect of enalapril. Based on these findings, we hypothesize that the patient’s persistent proteinuria can be attributed to hypoplastic kidney, whereas the resolution of hematuria indicated remission of C1qN. However, given the lack of a recent pathological evaluation, the extent to which C1qN itself contributed to the residual proteinuria remains unclear. Therefore, pathological re-evaluation will be considered if proteinuria worsens or if hematuria recurs.

## Conclusions

This case report highlights the long-term disease course in a pediatric patient with bilateral hypoplastic kidney and massive proteinuria who later developed hematuria and was subsequently diagnosed with C1qN over four years after disease onset. Despite the presence of high-risk factors and concerns regarding progressive renal dysfunction, our patient maintained stable kidney function for almost 10 years with enalapril monotherapy. This case demonstrates that asymptomatic C1qN with mild proliferative GN might have a favorable prognosis without the administration of steroids, even in cases of hypoplastic kidneys with renal dysfunction and massive proteinuria. Additionally, it highlights the importance of prompt pathological evaluation in guiding appropriate interventions.
